# Complete genome sequence of *Escherichia* Siphophage Serwaa

**DOI:** 10.1128/mra.01222-25

**Published:** 2026-05-29

**Authors:** Michael Acheampong Debrah, Michael Baffour Awuah, Annie Koh, Jolene Ramsey

**Affiliations:** 1 Department of Biology, Texas A&M University 14736, College Station, Texas, USA; 2 Center for Phage Technology, Texas A&M University 14736, College Station, Texas, USA; 3 Department of Biomedical Sciences, Texas A&M University 14736, College Station, Texas, USA; University of Maryland Baltimore, Baltimore, Maryland, USA

**Keywords:** bacteriophages, *Escherichia coli*, DNA sequencing, genomics

## Abstract

*Escherichia coli* 4s is a Gram-negative bacterium of the equine gastrointestinal tract microbiome. Here, we report the complete genome of 4s siphophage, Serwaa. The Serwaa genome is 62,144 bp with 42 of 85 proteins assigned a predicted function. Serwaa shares the highest similarity with other *Nonagviruses*.

## ANNOUNCEMENT

**Fig 1:**
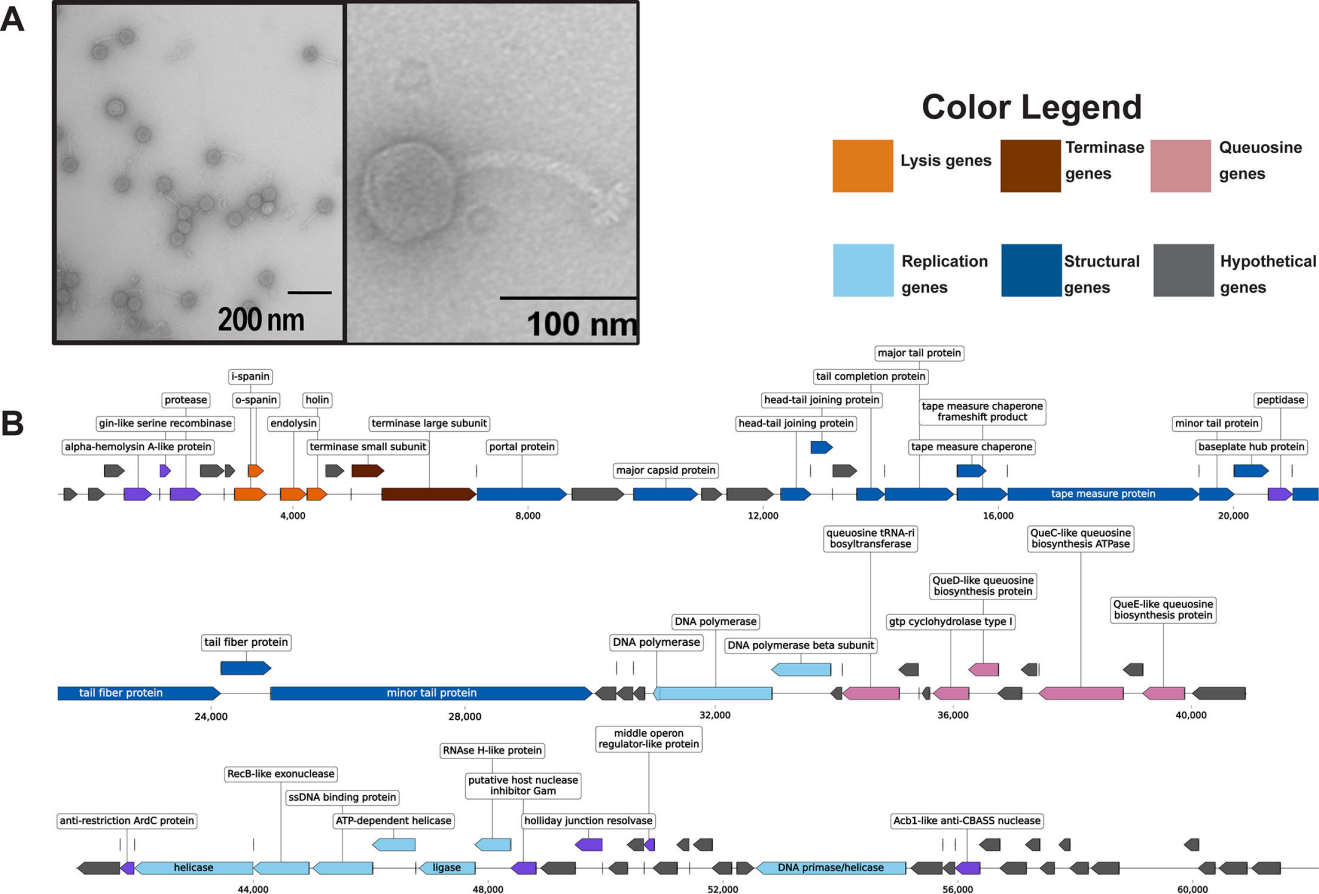
Morphology and genome map of the annotated *Escherichia* phage Serwaa. (**A**) Serwaa transmission electron micrograph shows an icosahedral head with flexible, noncontractile tails. (**B**) The protein-coding genes predicted in the Serwaa genome with their putative functional assignments are displayed. Gene group colors are assigned as lysis (orange), replication (sky blue), terminase (brown), structural (blue), queuosine (magenta), and hypothetical proteins of unknown function (gray).

*E. coli* are rod-shaped bacteria found in the large intestines of mammals (1, 2). The *E. coli* 4s strain is a dominant horse intestine commensal (3). Exploring the dynamism of phage infecting *E. coli* 4s will broaden our understanding of unique phage-bacterium interactions within microbiomes. Here, we describe a newly isolated *E. coli* 4s siphophage called Serwaa (Fig. 1).

Serwaa was isolated from surface soil near a pond outside Cameron, Texas (30°53′44.0″N 96°51′18.0″W) by enrichment on the *E. coli* 4s host cultured aerobically in Lysogeny Broth at 37°C. Briefly, soil was suspended in Lysogeny Broth at a 1:4 (wt/vol) ratio and then passed through a 0.22 µm filter. After overnight incubation of saturated *E. coli* 4s culture in the filtrate, phages were recovered from the supernatant. The phage was cultured by the soft agar overlay method (4). We visualized morphology using 2% (wt/vol) uranyl acetate negative staining for transmission electron microscopy at the Texas A&M University Microscopy and Imaging Center (5). The genomic DNA was extracted and purified as previously described using the Promega Wizard DNA clean-up system (6); then libraries were prepared with the Illumina DNA Prep tagmentation kit and sequenced at SeqCoast Genomics (Portsmouth, NH) with an Illumina NextSeq 2000 platform as 2 × 150 bp reads using a 300-cycle flow cell kit. Reads were demultiplexed using DRAGEN v3.10.12. The 781,976 reads were quality controlled using FastQC v0.11.9 (https://www.bioinformatics.babraham.ac.uk/projects/fastqc/). A single contig was assembled from untrimmed reads at 344× fold coverage using SPAdes v3.12.0 (7). The contig was confirmed complete by Sanger sequencing of the product amplified by PCR across the contig ends using manufacturer recommendations for Phusion polymerase with forward (5′-CGGACCTCAGTCCGCTGG-3′) and reverse (5′-GGCAGGAGCAGGGTAAGACG-3′) primers at a 61°C annealing temperature. Finally, the contig was reopened upstream of the last gene on the same coding strand as the terminase subunits, syntenic with many other *Nonagviruses*. All tools mentioned for annotation and assembly were used at default settings in the Center for Phage Technology European Union Galaxy instance (https://phage.usegalaxy.eu/) (8–11). GLIMMER v3.02 and MetaGeneAnnotator v1.0 were used to predict protein-coding genes (12, 13). No tRNA genes were detected using ARAGORN v1.2.36 (14). Functional annotations of protein-coding genes were predicted using BLAST 2.10.1, and conserved domains were detected using InterProScan v5.59-91.0 databases (11, 15). The TMHMM v2.0 database was used to find transmembrane proteins (16). Any conflicts were resolved by manual review.

Serwaa has a 62,144 bp genome with a 51% G + C content and a 92.4% coding density. Its genome is predicted to have 85 protein-coding genes with 42 assigned a predicted function (Fig. 1). After BLASTn sequence alignment, Serwaa showed the highest nucleotide sequence identity, at 80.4%, to Klebsiella phage vB_KppS-Raw (GenBank accession number LR880964.1). By VipTree proteome-based analysis, Serwaa is most similar to Salmonella phage SE1 (GenBank NC_042025.1) and other *Nonagviruses* (17).

## Data Availability

The sequence and associated data for phage Serwaa have been deposited under GenBank accession number PX021331, BioProject accession number PRJNA222858, SRA accession number SRR34773693, and BioSample accession number SAMN50231104.
